# Pregnant Women and Malaria Preventive Measures: A Case of Tamale Teaching Hospital, Ghana

**DOI:** 10.1155/2021/6150172

**Published:** 2021-12-07

**Authors:** Abdul Rauf Alhassan

**Affiliations:** Department of Surgery, Tamale Teaching Hospital, P.O. Box TL 16, Tamale, Ghana

## Abstract

**Background:**

In Saharan Africa, an estimated 25 million pregnancies are all at risk of malaria every year, with substantial morbidity and death effects for both the mother and the fetus.

**Aim:**

To investigate the use of malaria preventive measures among pregnant women patronizing antenatal services of Tamale Teaching Hospital. *Methodology*. This study was conducted using a descriptive cross-sectional survey of 250 participants. Data analysis was done with SPSS version 20. Graphs and tables were used to present the study data. Bivariate analysis was done using Chi-square use to determine the relationships and binary logistics regression used for identification of predictor variables.

**Results:**

The mean age of the study participants was 30.0 ± 4.5 years and most of them (73.0%) were within the age group of 25–35 years. Respondents' favorable knowledge, a favorable attitude, and favorable practice were 78.0%, 62.0%, and 57.6%, respectively. And the following variables were associated with malaria preventive practice: age of the respondent (*X*^2^ = 6.276, *P*=0.043), religion (*X*^2^ = 6.904, *P*=0.032), level of education (*X*^2^ = 41.482, *P* < 0.001), employment status (*X*^2^ = 20.533, *P* < 0.001), monthly income (*X*^2^ = 21.838, *P* < 0.001), and attitude level towards malaria prevention (*X*^2^ = 35.885, *P* < 0.001). Further analysis revealed educational level and attitude level as predictors of malaria preventive practice.

**Conclusion:**

This study recorded favorable knowledge, attitude, and practice with regards to malaria prevention among more than half of the study participants.

## 1. Introduction

Malaria is the biggest cause of mortality and other socioeconomic consequences in Ghana, owing to morbidity and its social, economic, and health consequences [1–3]. Malaria continues to have a severe influence on health and livelihood across the world, even though it is preventable and treated [4]. Malaria affects around 216 million people worldwide each year [5]. Malaria claimed the lives of around 445,000 people in 2016, with 91.0% of deaths occurring in Sub-Saharan Africa [5].

In Ghana, the National Malaria Control Programme (NMCP), which began operations in 1999, establishes the procedures for reducing malaria's impact across the country [6]. Malaria control treatments such as the use of insecticide-treated nets (ITNs), indoor residual spraying (IRS), and rapid diagnostic tests (RDTs), all of which are part of Ghana's core malaria control strategies, have made a substantial contribution to malaria control [7]. The World Health Organization (WHO) recommends a combination of tactics to battle malaria, including raising knowledge of the disease's causes and prevention, improving surveillance, and developing new vaccine control mechanisms [8].

Pregnant women in malaria-endemic areas are at a higher risk of getting the disease [9], with more severe consequences than their nonpregnant counterparts [10]. Malaria is a leading cause of death among newborns and children under the age of five, pregnant women, immunocompromised people, tourists, and migration from malaria-free areas. Pregnant women, their unborn babies, and children under the age of five are the most vulnerable to infection and negative effects [11]. Due to impaired and immature immunity in these groups, they are at greater risk [12]. Malaria while pregnancy is linked to negative health consequences such as maternal anemia, IUGR, and low birth weight babies. In Sub-Saharan Africa, low birth weight (less than 2500 g) is the primary cause of infant death [13].

In tropical and subtropical countries, malaria infection during pregnancy is a serious public health concern, posing a severe risk to both the pregnant woman and her fetus. According to the annual report by Tegegne et al. [14], there were around 25 million pregnant women at risk of malaria. Malaria-related deaths in pregnancy are estimated to occur 10,000 times per year in Sub-Saharan Africa, with severe maternal anemia being the most common cause [14]. According to the results of the current systematic review and meta-analysis in Africa, the pooled prevalence of malaria among pregnant women is greater than that in the general population [14]. In Sub-Saharan Africa, an estimated 25 million pregnancies are all at risk of malaria every year, with substantial morbidity and death effects for both the mother and the fetus [11, 12, 15]. In Ghana, *Plasmodium falciparum* is responsible for 80–90 percent of malaria-related morbidity, particularly among pregnant women and children under the age of five [16].

*Plasmodium falciparum* infection was found in one out of every five pregnant women (20.4%) visiting their first ANC clinic visit in a malaria-endemic district in Ghana's middle belt. The majority of the infections had a parasite count of fewer than 1000 parasites per liter and were associated with anemia [17]. A study in Northern Ghana involving Tamale Teaching Hospital, Tamale Central Hospital, Tamale West Hospital, and Bilpella Health Centre revealed the prevalence of malaria infection among pregnant women to be 14.1% using RDTs tests and 13.4% using PCR [18].

Malaria during pregnancy is still a serious public health issue in Africa and Ghana, and it has been linked to several pregnancy-related problems. Updated and accurate data on the risk factors are required to design effective and timely health policies for the prevention and control of malaria and anemia in pregnancy. Therefore, this current study seeks to identify malaria preventive measures among pregnant women patronizing the antenatal services of Tamale Teaching Hospital (TTH), Ghana.

## 2. Methodology

### 2.1. Study Design and Site

A descriptive cross-sectional survey was used for this study, using quantitative data for analysis. The study took place in the antenatal clinic of Tamale Teaching Hospital. In 1974, Tamale Teaching Hospital, then Tamale Regional Hospital, was founded. In 2005, the hospital was promoted to the status of Teaching Hospital under Act 525 of the Ghana Health Service and Teaching Hospitals Act of 1996. Tamale Teaching Hospital is the country's third teaching hospital in Ghana. It serves as a referral center for the northern areas of Ghana.

### 2.2. Study Participants

The population under this study was all pregnant women visiting the antenatal clinic of Tamale Teaching Hospital. From survey at the antenatal clinic of TTH it was revealed that the daily average antenatal attendance at the clinic is 50 pregnant women. And at least one week is the required interval between visits for pregnant women depending on the gestational weeks of the pregnancy. Hence, the population was calculated for five days (one week minus Saturday and Sunday) to avoid duplication of subjects in the total population. The total population for this study was 50 × 50 = 250. This study recruited all 250 estimated population.

### 2.3. Data Collection Tool and Procedure

The duration for the data collection was one week (Monday to Friday) based on the clinic days of the antenatal clinic of TTH. Pregnant women with a gestational age of 16 weeks and above were selected for this study to measure IPTp-SP use, which starts after 16 weeks of gestation. The self-administrable questionnaire was used and respondents who cannot read were interviewed using the questionnaire.

### 2.4. Study Variables

In this study, the major dependent variable was respondents' malaria infection prevention practice level, whereas the independent factors were respondents' demographics, knowledge of malaria infection and prevention, and attitude toward malaria infection prevention.

The average scores for knowledge, attitude, and practice were applied as the cut-off point for the classification of knowledge, attitude, and practice levels taking reference from an earlier similar study [19].

### 2.5. Data Analysis

The Statistical Package for the Social Sciences (SPSS) version 20 was used for data entry and analysis. To allow for quantitative analysis, responses to categorical variables were coded. To ensure data accuracy and preserve the study's good validity, data cleaning was performed. Age was given as a continuous variable with a standard deviation. Categorical variables, such as gender, were presented as a percentage. The study data was presented using graphs and tables. The association was determined using Chi-square analysis and binary logistics regression applied for predictors' malaria prevention practice.

### 2.6. Ethical Consideration

The process of data collection started immediately after a certificate of authorization to research the hospital was granted by the research department of Tamale Teaching Hospital. Participants' consent was obtained to answer the questionnaire, the information provided was treated with confidentiality, and participants were given access to the results of the study. Any form of physical or psychological harm towards participants was avoided. All sources for information used in this research were duly acknowledged to avoid any form of plagiarism.

## 3. Results

### 3.1. Demographic Characteristics of Respondents

The study recorded a 100 percent response rate, the mean age of the study participants was 30.0 ± 4.5 years, and most of them (73.0%) were within the age group of 25–35 years. The gestational age of pregnancy in terms of weeks was 28–38 weeks for just a little above half (52.8%) of the participants. The religious affiliation of most of (69.4%) of the participants was Islam and 95.6% of the study participants were married. In terms of employment, 44.2% were self-employed, and the majority (77.2%) of the respondents were earning less than 1000 Ghana cedis as their monthly income (Table 1).

### 3.2. Pregnant Women Knowledge on Malaria Preventive Measures

On respondent knowledge assessment, almost (99.6%) all have heard of malaria before and the dominant source of this information was school per 41.4% of the respondents. Most (97.2%) of the respondents indicated that malaria can be transmitted through the bite of an infected mosquito. About 29.0% of the respondents indicated that malaria can be transmitted by drinking contaminated water and 10.0% indicated that malaria can be transmitted by coming into close contact with a malaria patient. The majority (95.2%) of the respondents indicated the fact that sleeping under bed nets can prevent malaria. 71.9% of the respondents indicated wearing long-sleeved clothing during the night to prevent malaria. Also, the majority of the respondents agreed with the following means of preventing malaria infection: spraying insecticide can prevent malaria (96.8%), trimming bushes around the house can prevent malaria (94.8%), cleaning dark corners in the house can prevent malaria (94.4%), elimination of stagnant water around the house prevents the breeding of mosquitos (96.0%), and netting doors and windows prevents malaria attack (96.0%). Most of the respondents (92.4%) knew that IPTP drugs should be taken twice after 16 weeks of pregnancy to prevent malaria attacks. Overall, most (78.0%) of the respondents had a favorable knowledge level on malaria prevention (Table 2).

### 3.3. Level of Attitude Pregnant Women Have toward Malaria Preventive Measures

The majority (95.2%) of respondents disagreed that people should avoid close contact with people infected with malaria. About 59.8% disagreed that one can recover spontaneously from malaria without any treatment. However, 94.0% of the respondents agreed that they can buy antimalaria drugs from the drug shop/pharmacy to treat themselves when they get malaria. And most (96.0%) will seek advice or treatment when they get malaria. Most (98.8%) of the respondents agreed with the statement “the best way to prevent me from getting malaria is to avoid getting mosquito bites.” A good number (59.8%) of the respondents believe that sleeping under a mosquito net during the night can be avoided because of heat. The opinion of most of the respondents (83.5%) was that children and pregnant women are most at risk of malaria, and most (98.0%) of the respondents agreed that anyone can still get malaria. The majority (98.8%) of the participant pregnant women agree that they might be at a greater risk of getting malaria if they work and sleep overnight in the garden or forest. It is dangerous when malaria medicine is not taken completely as agreed by most (83.5%) of the respondents. Meanwhile, the majority (83.9%) of the pregnant women agreed that it was difficult to take antenatal malaria prophylaxis drug (SP). Overall, most (62.0%) of the respondents had a favorable attitude level towards malaria prevention (Table 3).

### 3.4. Pregnant Women Practice Malaria Preventive Measures

In terms of insecticide-treated nets (ITNs) ownership, most (83.2%) of the respondents had ITNs at their homes. And the majority (62.4%) of the respondents had them free of charge from campaigns. About 82.3% of the respondents were sleeping under ITNs to prevent malaria. The practice of wearing long-sleeved clothing to prevent malaria was practiced by about half (52.8%) of the respondents. Most (96.8%) of the respondents answered yes to the question: Do you use insecticide spray or mosquito coil to prevent malaria? Most (76.8%) were trimming bushes around their homes to prevent malaria. Other environmental practices by most of the respondents were cleaning dark corners in the house to prevent malaria (69.2%), eliminating stagnant water around the house to prevent the breeding of mosquitos (72.6%), and putting nets on the doors and windows to prevent malaria attack (78.3%). And almost all (98.0%) were taking IPTP SP drugs to prevent malaria attacks. Overall, 57.6% of the respondents had favorable malaria prevention practice levels on malaria prevention (Table 4).

### 3.5. Factors Associated with Pregnant Women Practice Level on Malaria Prevention

To identify variables that were associated with pregnant women's practice of malaria prevention, chi-square analysis was used. And the following variables were associated with significance: age of the respondent (*X*^2^ = 6.276, *P*=0.043), religion (*X*^2^ = 6.904, *P*=0.032), level of education (*X*^2^ = 41.482, *P* < 0.001), employment status (*X*^2^ = 20.533, *P* < 0.001), monthly income (*X*^2^ = 21.838, *P* < 0.001), and attitude level towards malaria prevention (*X*^2^ = 35.885, *P* < 0.001). However, the remaining factors such as marital status, gestational weeks of pregnancy, and knowledge level on malaria prevent were not associated with malaria prevention practice level (Table 5).

Socioeconomic variables with significance at the bivariate analysis stage were further modeled using binary logistics regression. And the variables with significant prediction were educational level and attitude level towards malaria prevention. Those with tertiary educational levels were 6.8 times more likely to practice malaria prevention as compared to those without education (AOR = 7.8, 95%, C.I. = 1.5–41.7). Also, those with favorable attitude levels towards malaria prevention were 2.6 times more likely to practice malaria prevention as compared to those with unfavorable attitude (AOR = 3.6, 95%, C.I. = 1.8–7.0) (Table 6).

## 4. Discussion

The majority of the pregnant women had favorable knowledge of malaria preventive measures. This study result is good and can be considered high as compared to earlier studies [20–22].

On respondent knowledge assessment, almost all of the respondents have heard of malaria before and the dominant source of this information was school. Most of the respondents indicated that malaria can be transmitted through the bite of an infected mosquito, contrary to about 29.0% of the respondents who indicated that malaria can be transmitted by drinking contaminated water and 10.0% who indicated that malaria can be transmitted by coming into close contact with a malaria patient. Malaria is not an infectious disease and cannot be transmitted through drinking contaminated water; the source of transmission is through the bite of an infected mosquito [23].

The majority of the respondents knew that sleeping under bed nets can prevent malaria and wearing long-sleeved clothing during the night can prevent malaria. Also, the majority of the respondents agreed with the following means of preventing malaria infection: spraying insecticide can prevent malaria, trimming bushes around the house can prevent malaria, cleaning dark corners in the house can prevent malaria, elimination of stagnant water around the house prevent the breeding of mosquitos, and netting doors and windows can prevent malaria attack. This is a similar or even a little higher level than that of an earlier study in which most participants knew preventive measures of malaria [24]. However, a study in Cameroun reported lower knowledge for preventive measures among pregnant women [25].

From the second trimester onwards, the World Health Organization recommends administering IPTp-SP at each ANC check, with a one-month interval between visits [26]. And the good thing is that in this present study most of the respondents knew that IPTP drugs should be taken twice after 16 weeks of pregnancy to prevent malaria attacks.

From a study in Ethiopia, a little more than half of the pregnant women (51.1%) had a favorable view of malaria and ITNs [19]. This is reported high in this current study as 62.0% of the pregnant women had a favorable attitude towards malaria prevention. Adibe et al. study in Nigeria recorded even less (49.0%) percentage of pregnant women with a positive attitude towards malaria prevention [27].

Malaria is not an infectious disease and cannot be transmitted through body contact, and the source of transmission is through the bite of an infected mosquito [23]. The study respondents had a positive attitude in this regard as most of them disagreed that people should avoid close contact with people infected with malaria. Meanwhile, in earlier studies, the respondents viewed malaria can be transmitted from personal contact [27, 28].

In Ghana, recommended protocol for malaria management is Test, Treat, and Track (T3), which means that testing for malaria before treatment is encouraged whenever possible [29]. Meanwhile, study participants' attitude towards malaria treatment was unfavorable as a good number agreed that one can recover spontaneously from malaria without any treatment and most of them agreed that they can buy antimalaria drugs from the drug shop/pharmacy to treat themselves when they get malaria. This study finding is not different when compared to an earlier Nigeria study [27]. However, most of the respondents will seek advice or treatment when they get malaria. Also, in an earlier study, a significant number of the respondents stated that they could treat themselves if they got malaria and thought they could recover from malaria on their own [28].

The best way to avoid getting malaria is to avoid getting mosquito bites [30]. However, in this present study, the attitude towards ITNs was poor as about 59.8% of the respondent believe that sleeping under a mosquito net during the night can be avoided because of heat.

Malaria is a leading cause of death among children under the age of five and pregnant women and they are most vulnerable to malaria infection and negative effects [11]. In this present study, most of the respondents were aware of the view that children and pregnant women are most at risk of malaria and that anyone can still get malaria.

From the second trimester onwards, the World Health Organization recommends administering IPTp-SP at each ANC check, with a one-month interval between visits [26]. This attitude towards this in this present study is not good as the majority of the pregnant women agreed that it is difficult to take antenatal malaria prophylaxis drug (SP).

Most (57.6%) had favorable practices concerning malaria prevention, and this is low as compared to an earlier study [31]. Adibe et al. study in Nigeria recorded even less (41.0%) percentage of pregnant with favorable self-practice towards malaria prevention [27].

In terms of insecticide-treated nets (ITNs) ownership, most of the respondents had ITNs at their homes, the majority of the respondent had them free of charge from campaigns and were sleeping under ITNs to prevent malaria. This is similar to a report from a Tanzanian study [28]. And in a Nigerian study the use of ITN among pregnant women was very low [27].

The other preventive practices reported practiced by most respondents in this present study included wearing long-sleeved clothing, use of insecticide spray or mosquito coil, trimming bushes around their homes, cleaning of dark corners, and netting of doors and windows. These practices were reported mostly low in earlier studies [19, 28]. Even though IPTp-SP coverage has grown in recent years, less than a third of pregnant women in moderate to high endemic areas receive three or more doses of the vaccine [23]. Meanwhile, in this current study, almost all (98.0%) were taking IPTP SP drugs to prevent malaria attacks.

Also, in another similar study in Cameroun, maternal age, marital status, level of education, and occupation were significantly statistically associated with malaria preventive measures in pregnant women [32]. In this present study, the following socioeconomic variables have significance with malaria prevention practice: the age of the respondent, religion, level of education, employment status, monthly income, and attitude level towards malaria prevention. However, in the same study, marital status was associated with malaria preventive measures in pregnant women [32]. And in this present study marital status did not make a significant difference with malaria prevention practice.

Similar to Bhalla et al. study, educational level of pregnant women was associated with malaria preventive measures, but economic status and knowledge were not associated with preventive measures [33]. Furthermore, in this present study, those with tertiary educational levels were 6.8 times more likely to practice malaria prevention as compared to those without education. Also, in another similar study, pregnant women with secondary and above educational levels were 22.2 times more likely to practice malaria prevention as compared to illiterates [19].

Attitude is expected to influence many practice behavior [34]. This is confirmed in this present study as those with favorable attitude levels towards malaria prevention were 2.6 times more likely to practice malaria prevention as compared to those with unfavorable attitudes. In a similar study, attitude level did not make a significant difference in malaria prevention practice among pregnant women [19].

This study has some limitations. Since respondents were selected primarily from prenatal clinics, the results may not have reflected the thoughts of pregnant women in marginalized rural communities who cannot afford antenatal care services at TTH. Furthermore, this study acknowledges that the hospital base research is not representative of the general population.

## 5. Conclusion

About 4 out of 5 pregnant women had favorable knowledge with regards to malaria preventive measures. Most of the respondents knew that IPTP drugs should be taken twice after 16 weeks of pregnancy to prevent malaria attacks.

An average number of the respondents showed a favorable attitude towards malaria preventive measures. The majority of the pregnant women were of the view that it is difficult to take antenatal malaria prophylaxis drugs (SP).

Malaria preventive practice was favorable among an average number of respondents. And predictors of practice were educational level and attitude level.

### 5.1. Recommendation

This study revealed a poor attitude toward the use of the insecticide-treated net. Nurses and midwives ought to intensify their education on malaria prevention, especially with pregnant women, about the use of insecticide-treated net usage. Future research to identify predictors of ITNs usage among pregnant women in the northern region is recommended.

## Figures and Tables

**Table 1:** Demographic characteristics of the respondents.

		Frequency (250)	Percentage (%)
Age group	18–25 years	41	16.5
	26–35 years	181	73.0
	36–55 years	26	10.5

Religion	Islam	172	69.4
	Christianity	74	29.8
	Traditional	2	0.8

Marital status	Married	238	95.6
	Single	7	2.8
	Widow	3	1.2
	Divorce	1	0.4

Level of education	None	28	11.2
	Primary	72	28.8
	Secondary	68	27.2
	Tertiary	82	32.8

Employment status	Unemployed	61	24.5
	Self-employed	110	44.2
	Government-employed	78	31.3

Monthly income	<1000 GH	176	77.2
	2000–3000 GH	40	17.5
	>3000 GH	12	5.3

Gestation weeks grouped	16–27 weeks	117	47.2
	28–38 weeks	131	52.8

Source: field survey (2021).

**Table 2:** Respondents' knowledge on malaria preventive measures.

		Frequency (250)	Percentage (%)
Have you heard of malaria?	No	1	0.4
	Yes	249	99.6

If 1, from where?	Family member	47	18.9
	Media	31	12.4
	Poster	15	6.0
	School	103	41.4
	Church or Mosque	12	4.8
	Health facility	41	16.5

Malaria can be transmitted from the bite of a mosquito infected with malaria	No	7	2.8
	Yes	239	97.2

Malaria can be transmitted by coming into close contact with a malaria patient	No	224	90.0
	Yes	25	10.0

Malaria can be transmitted by drinking contaminated water	No	176	71.0
	Yes	72	29.0

Sleeping under bed nets can prevent malaria	No	12	4.8
	Yes	236	95.2

Wearing long-sleeved clothing during the night can prevent malaria	No	70	28.1
	Yes	179	71.9

Spraying insecticides can prevent malaria	No	8	3.2
	Yes	242	96.8

Trimming bushes around the house can prevent malaria	No	13	5.2
	Yes	235	94.8

Cleaning dark corners in the house can prevent malaria	No	14	5.6
	Yes	236	94.4

Do you know that the IPTP drug should be taken twice after 16 weeks of pregnancy to prevent malaria attacks?	No	19	7.6
	Yes	231	92.4

Does eliminating stagnant water around the house prevent the breeding of mosquitos?	No	10	4.0
	Yes	238	96.0

Does netting doors and windows prevent malaria attacks?	No	10	4.0
	Yes	239	96.0

Knowledge level on malaria prevention	Unfavourable	55	22.0
	Favourable	195	78.0

Source: field survey (2021).

**Table 3:** Respondents' attitude towards malaria preventive measures.

		Frequency (250)	Percentage (%)
If someone has malaria, people should avoid having close contact with him or her	Agree	7	2.8
	Disagree	238	95.2
	Neutral	5	2.0

I think that one can recover spontaneously from malaria without any treatment	Agree	50	20.1
	Disagree	149	59.8
	Neutral	50	20.1

I can buy antimalaria drugs from the drug shop/pharmacy to treat myself when I get malaria	Agree	233	94.0
	Disagree	4	1.6
	Neutral	11	4.4

I think the best way to prevent myself from getting malaria is to avoid getting mosquito bites	Agree	246	98.8
	Disagree	3	1.2
	Neutral	0	0.0

I will seek advice or treatment when I get malaria	Agree	239	96.0
	Disagree	7	2.8
	Neutral	3	1.2

I believe sleeping under a mosquito net during the night can be avoided because of heat	Agree	149	59.8
	Disagree	78	31.3
	Neutral	22	8.8

In my opinion, mostly children and pregnant women are at risk of malaria	Agree	208	83.5
	Disagree	13	5.2
	Neutral	28	11.2

I might be at a greater risk of getting malaria if I work and sleep overnight in the garden or forest	Agree	245	98.8
	Disagree	2	0.8
	Neutral	1	0.4

I am sure that anyone can get malaria	Agree	243	98.0
	Disagree	1	0.4
	Neutral	4	1.6

I think that it is dangerous when malaria medicine is not taken completely	Agree	207	83.5
	Disagree	8	3.2
	Neutral	33	13.3

I think that I should go to the health center/clinic to have my blood tested as soon as I suspect that I have suffered from malaria	Agree	236	95.2
	Disagree	2	0.8
	Neutral	10	4.0

It is difficult to take antenatal malaria prophylaxis drug (SP)	Agree	209	83.9
	Disagree	19	7.6
	Neutral	21	8.4

Attitude level toward malaria preventive measures	Unfavourable	95	38.0
	Favourable	155	62.0

Source: field survey (2021).

**Table 4:** Respondents practice of malaria preventive measures.

		Frequency (250)	Percentage (%)
Do you own insecticide-treated nets (ITNs) at home?	No	42	16.8
	Yes	208	83.2

Source of ITN	Buying at shops	24	10.9
	Free of charge from campaigns	138	62.4
	Subsidized price from health facility	59	26.7

Do you sleep under bed nets to prevent malaria?	No	44	17.7
	Yes	205	82.3

Do you wear long-sleeved clothing in the evening to prevent malaria?	No	118	47.2
	Yes	132	52.8

Do you use insecticide spray or mosquito coil to prevent malaria?	No	8	3.2
	Yes	241	96.8

Do you trim bushes around the house to prevent malaria?	No	58	23.2
	Yes	192	76.8

Do you clean dark corners in your house to prevent malaria?	No	77	30.8
	Yes	173	69.2

Do you take IPTP SP drug after 16 weeks of pregnancy to prevent malaria attacks?	No	5	2.0
	Yes	245	98.0

Do you eliminate stagnant water around the house to prevent the breeding of mosquitos?	No	68	27.4
	Yes	180	72.6

Do you put net on your doors and windows to prevent malaria attacks?	No	54	21.7
	Yes	195	78.3

Malaria prevention practice level	Unfavourable	106	42.4
	Favourable	144	57.6

Source: field survey (2021).

**Table 5:** Chi-square analysis for factors associated with pregnant women practice level on malaria prevention.

		Malaria preventive practice level				Test statistic
		Unfavourable		Favourable		
Age group	18–25 years	23	56.1%	18	43.9%	**X** ^**2**^ = **6.276**
	26–35 years	68	37.6%	113	62.4%	**P**=0.043
	36–55 years	14	53.8%	12	46.2%	

Religion	Islam	81	47.1%	91	52.9%	**X** ^**2**^ = **6.904**
	Christianity	23	31.1%	51	68.9%	**P**=0.032
	Traditional	0	0.0%	2	100.0%	

Marital status	Married	99	41.6%	139	58.4%	*X* ^2^ = 0.723
	Single	6	54.5%	5	45.5%	*P*=0.395

Level of education	None	24	85.7%	4	14.3%	
	Primary	37	51.4%	35	48.6%	**X** ^**2**^ = **41.482**
	Secondary	29	42.6%	39	57.4%	**P** < 0.001
	Tertiary	16	19.5%	66	80.5%	

Employment status	Unemployed	39	63.9%	22	36.1%	**X** ^**2**^ = **20.533**
	Self-employed	47	42.7%	63	57.3%	**P** < 0.001
	Government-employed	20	25.6%	58	74.4%	

Monthly income	<1000 GH	89	50.6%	87	49.4%	**X** ^**2**^ = **21.838**
	2000–3000 GH	8	20.0%	32	80.0%	**P** < 0.001
	>3000 GH	0	0.0%	12	100.0%	

Gestation weeks grouped	16–27 weeks	50	42.7%	67	57.3%	*X* ^2^ = 0.0
	28–38 weeks	56	42.7%	75	57.3%	*P*=0.998

Malaria preventive knowledge level	Unfavourable	28	50.9%	27	49.1%	*X* ^2^ = 2.090
	Favourable	78	40.0%	117	60.0%	*P*=0.148

Attitude level toward malaria prevention	Unfavourable	63	66.3%	32	33.7%	**X** ^**2**^ = **35.885**
	Favourable	43	27.7%	112	72.3%	**P** < 0.001

Source: field survey (2021).

**Table 6:** Binary logistics for socioeconomic predictors of malaria preventive practice.

	Wald	Sig.	AOR	95% C.I. for AOR	
				Lower	Upper
18–25 years	Reference				
26–35 years	0.173	0.678	0.787	0.255	2.432
36–55 years	0.615	0.433	0.560	0.132	2.385

Islam	Reference				
Christianity	1.605	0.205	1.584	0.777	3.227
Traditional	0.000	0.999	327974397.959	0.000	

None	Reference				
Primary	2.112	0.146	2.893	0.690	12.127
Secondary	2.870	0.090	3.550	0.820	15.371
Tertiary	5.782	**0.016**	**7.806**	**1.462**	**41.674**

Unemployed	Reference				
Self-employed	2.170	0.141	2.344	0.755	7.281
Government-employed	1.970	0.160	2.566	0.688	9.561

<1000 GH	Reference				
2000–3000 GH	0.108	0.743	1.226	0.363	4.133
>3000 GH	0.000	0.999	417621634.591	0.000	

Unfavourable attitude level	Reference				
Favourable attitude level	13.699	**0.000**	**3.576**	**1.821**	**7.020**
Constant	10.111	0.001	0.083		

Source: field survey (2021).

## Data Availability

The data supporting the findings of this study are available from the corresponding author upon request.
